# Melatonin Accelerates Osteoporotic Bone Defect Repair by Promoting Osteogenesis–Angiogenesis Coupling

**DOI:** 10.3389/fendo.2022.826660

**Published:** 2022-02-22

**Authors:** Sheng Zheng, Chunhao Zhou, Han Yang, Junhua Li, Ziyu Feng, Liqing Liao, Yikai Li

**Affiliations:** ^1^ School of Traditional Chinese Medicine, Southern Medical University, Guangzhou, China; ^2^ Department of Orthopedics-Spine Surgery, Nanfang Hospital, Southern Medical University, Guangzhou, China

**Keywords:** melatonin, osteoporosis, bone defect repair, osteogenesis–angiogenesis coupling, bone marrow mesenchymal stem cells

## Abstract

Previous studies have revealed that melatonin could play a role in anti-osteoporosis and promoting osteogenesis. However, the effects of melatonin treatment on osteoporotic bone defect and the mechanism underlying the effects of melatonin on angiogenesis are still unclear. Our study was aimed to investigate the potential effects of melatonin on angiogenesis and osteoporotic bone defect. Bone marrow mesenchymal stem cells (BMSCs) were isolated from the femur and tibia of rats. The BMSC osteogenic ability was assessed using alkaline phosphatase (ALP) staining, alizarin red S staining, qRT-PCR, western blot, and immunofluorescence. BMSC-mediated angiogenic potentials were determined using qRT-PCR, western blot, enzyme-linked immunosorbent assay, immunofluorescence, scratch wound assay, transwell migration assay, and tube formation assay. Ovariectomized (OVX) rats with tibia defect were used to establish an osteoporotic bone defect model and then treated with melatonin. The effects of melatonin treatment on osteoporotic bone defect in OVX rats were analyzed using micro-CT, histology, sequential fluorescent labeling, and biomechanical test. Our study showed that melatonin promoted both osteogenesis and angiogenesis *in vitro*. BMSCs treated with melatonin indicated higher expression levels of osteogenesis-related markers [ALP, osteocalcin (OCN), runt-related transcription factor 2, and osterix] and angiogenesis-related markers [vascular endothelial growth factor (VEGF), angiopoietin-2, and angiopoietin-4] compared to the untreated group. Significantly, melatonin was not able to facilitate human umbilical vein endothelial cell angiogenesis directly, but it possessed the ability to promote BMSC-mediated angiogenesis by upregulating the VEGF levels. In addition, we further found that melatonin treatment increased bone mineralization and formation around the tibia defect in OVX rats compared with the control group. Immunohistochemical staining indicated higher expression levels of osteogenesis-related marker (OCN) and angiogenesis-related markers (VEGF and CD31) in the melatonin-treated OVX rats. Then, it showed that melatonin treatment also increased the bone strength of tibia defect in OVX rats, with increased ultimate load and stiffness, as performed by three-point bending test. In conclusion, our study demonstrated that melatonin could promote BMSC-mediated angiogenesis and promote osteogenesis–angiogenesis coupling. We further found that melatonin could accelerate osteoporotic bone repair by promoting osteogenesis and angiogenesis in OVX rats. These findings may provide evidence for the potential application of melatonin in osteoporotic bone defect.

## Introduction

Osteoporosis, as the most frequent bone disease, results in reduced bone strength. The main characteristics include lower bone mineral density (BMD) and bone mass, impaired bone quality, and abnormal micro-architecture (1–3). Osteoporosis is a common and age-related bone disease throughout the world, affecting more than 20 million individuals (4), which causes bone fragility and fractures (2). It has a major influence on individuals associated with high morbidity and mortality (5). As a global health concern, osteoporosis can affect both sexes and all races, dramatically increasing the social and economic burden worldwide.

Osteoporosis has been recognized as an increased risk of bone fracture and bone defect healing. Rodent studies showed that osteoporosis could cause a striking reduction in the callus size of bone fracture and bone defect, BMD, and mechanical strength (6). Previous studies showed that the healing time of bone fractures or bone defects was significantly longer in patients with osteoporosis than in healthy people (7–10). Maintaining osteogenesis and angiogenesis is crucial for osteoporotic bone regeneration. Ding et al. found that reduced local blood supply to the tibial metaphysis may be associated with ovariectomy-induced osteoporosis (11). In a rat osteoporotic model, new bone trabeculae is arranged in an irregular and loose fashion, indicating the poor bone quality of newly formed bone (12). For patients with osteoporotic bone defect, osteoclast activity is enhanced and bone resorption proceeds at a faster rate than that of bone formation. In addition, the ability of new bone formation is decreased, and bone defect healing is significantly delayed compared with normal bone defect (13). Thus, the treatment is more difficult than that of normal bone defect. In the face of such a severe health problem, how to find more therapeutic strategies and ideal drugs has become an urgent problem to be solved.

Melatonin, synthesized from serotonin in the pineal gland, is a signal molecule that modulates the biological circadian rhythms in humans (14). Except for the pineal gland, melatonin can also be synthesized locally in the bone marrow. Increasing evidence demonstrates that melatonin may play a critical role in bone metabolism. Melatonin, the synthesis of which decreases with aging, is considered to be involved in age-related bone loss and osteoporosis (15, 16). In bone, two types of membrane-bound melatonin receptors, including MT1 and MT2, have been identified and can be expressed in both osteoblasts and osteoclasts (17). The level of melatonin in the bone marrow was twice that of plasma at night (18), suggesting that it may be related to bone metabolism. Multiple studies have revealed that melatonin could promote osteoblast proliferation and differentiation, inhibit osteoclast activity, maintain the steady-state of bone metabolism, and thus play a role in anti-osteoporosis (19–22). Zhang et al. demonstrated that melatonin could restore the osteoporosis-impaired osteogenic potential of bone marrow mesenchymal stem cells (BMSCs) and alleviate bone loss through the HGF/PTEN/Wnt/beta-catenin axis (23). Dong et al. showed that melatonin treatment could upregulate the expressions of neuropeptide Y and its receptor Y1 and promote mesenchymal stem cell proliferation and migration (24). Thus, it indicates that melatonin may be a potential biomolecule for osteoporosis and its related bone defect.

Moderate osteogenesis and angiogenesis is involved in both bone repair and fracture healing (25). Currently, accumulating evidence has indicated the associations between melatonin and osteogenesis. However, few studies have been conducted to research the relationship between melatonin and angiogenesis. Ramírez-Fernández et al. observed that only the melatonin group showed a significantly increased number of blood vessels compared to the control group in a bone defect rabbit model (26). However, the mechanism underlying the effects of melatonin on angiogenesis was not clarified. Thus, how melatonin affects angiogenesis and what its effects are on osteoporotic bone defect are still unclear. The purpose of this study was to evaluate the potential effects of melatonin on angiogenesis and osteoporotic bone defect, which may provide evidence for the potential application of melatonin in osteoporosis and osteoporotic bone defect.

## Materials and Methods

### Isolation and Culture of Rat BMSCs

BMSC isolation was performed as previously described (27, 28). BMSCs were harvested from the bone marrow of femurs and tibias in 2-week-old Sprague–Dawley (SD) rats. The rats were euthanized and sterilized in 75% ethanol for 15 min. BMSCs were flushed out by an injection of alpha modified Eagle’s minimum essential medium (α-MEM; HyClone, USA) using a 5-ml syringe fitted with a 25-gauge needle under sterile conditions. After centrifugation, the BMSCs were cultured in α-MEM, which was supplemented with 10% fetal bovine serum (Gibco, USA) and 1% penicillin/streptomycin (Gibco, USA). The BMSCs between passages 3 and 5 were used in the following experiments. All of the above-mentioned cells were cultured at 37°C in a humidified atmosphere containing 5% CO_2_.

### Cell Proliferation Assay

BMSC proliferation was detected using Cell Counting kit-8 (CCK-8; Dojindo, Kumamoto, Japan) following the manufacturer’s instructions. Specifically, the BMSCs were seeded with a density of 2,000 cells per well in a 96-well plate and cultured in complete medium containing melatonin with various gradient concentrations (10 nM, 100 nM, 1 μM, and 10 μM) for various durations (1, 3, 5, and 7 days). Melatonin (purity >99%, cat. no. S20287) was purchased from Yuanye Bio-Technology Co., Ltd. (Shanghai, China). The untreated wells served as the control group. Then, each well was subjected to a 10-μl CCK-8 solution, and the cells were incubated at 37°C for 1 h. Then, the optical density was measured at 450 nm using a microplate reader (Thermo, USA).

### Osteoblastic Determination and Mineralization Assessment

BMSCs were seeded in a 24-well plate with a density of 2 × 10^4^ cells per well. The medium was replaced with an osteogenic medium (complete α-MEM containing 10 nM dexamethasone, 50 μM ascorbic acid, and 10 mM β-glycerophosphate) after reaching over 80% confluence. For treatment, melatonin with various gradient concentrations (10 nM, 100 nM, and 1 μM) was added into the medium. The untreated wells served as the control group. Then, protein was extracted, and the supernatant liquid was harvested after 7 days of osteogenic induction for western blotting analysis and enzyme-linked immunosorbent assay (ELISA) test of vascular endothelial growth factor (VEGF), respectively. Alkaline phosphatase (ALP) activity was determined at day 7 of differentiation using ALP Staining Kit (cat. no. P0321S, Beyotime Biotechnology, China). The mineralization of the calcium nodule was detected on the 14th day using alizarin red S (ARS) solution (cat. no. G1452, Solarbio Science & Technology, China). The absorbance at 405 nm for ALP and 560 nm for ARS staining was detected using a microplate reader (Thermo, USA).

### Quantitative Real-Time PCR

Prior to PCR, total RNA was extracted using RNA Purification Kit (EZBioscience, USA). The RNA was reverse-transcribed by 500 ng of total RNA from each sample using Reverse Transcription Kit (EZBioscience, USA). Next, the cDNA was amplified with SYBR Green qPCR Master Mix (EZBioscience, USA). Data were analyzed, and the relative expression levels were calculated by the 2^-ΔΔCT^ method. Housekeeping gene glyceraldehyde-3-phosphate dehydrogenase (GAPDH) was used for normalization. All reactions were carried out with three biological replicates, and each analysis consisted of three technical replicates. The primer sequences were designed by Oligo 7.0 software and are shown in **Table 1**.

**Table 1:** Real-time PCR primer sequences used in the study.

Gene	Forward primer (5′–3′)	Reverse primer (5′–3′)
ALP	CCGCAGGATGTGAACTACT	GGTACTGACGGAAGAAGGG
OCN	CAGACAAGTCCCACACAGCA	CCAGCAGAGTGAGCAGAGAGA
RUNX2	ACTTCCTGTGCTCGGTGCT	GACGGTTATGGTCAAGGTGAA
OSX	GGAAAAGGAGGCACAAAGAA	CAGGGGAGAGGAGTCCATT
VEGF	CACGACAGAAGGGGAGCAGAAAG	GGCACACAGGACGGCTTGAAG
Ang-2	GAAGAAGGAGATGGTGGAGAT	CGTCTGGTTGAGCAAACTG
Ang-4	GCTCCTCAGGGCACCAAGTTC	CACAGGCGTCAAACCACCAC
GAPDH	ATGGCTACAGCAACAGGGT	TTATGGGGTCTGGGATGG

ALP, alkaline phosphatase; OCN, osteocalcin; RUNX2, runt-related transcription factor 2; OSX, osterix; VEGF, vascular endothelial growth factor; Ang-2, angiopoietin-2; Ang-4, angiopoietin-4; GAPDH, glyceraldehyde-3-phosphate dehydrogenase.

### Western Blotting

Total protein was extracted by RIPA buffer (Beyotime Biotechnology, China), containing protease and phosphatase inhibitors (Sigma-Aldrich, USA), for 30 min at 4°C. The cell lysates were cleared by centrifugation, and the protein concentration was determined using the bicinchoninic acid quantification kit (Beyotime Biotechnology, China). Furthermore, 30 μg protein was electrophoresed with 10% SDS-PAGE electrophoresis (Beyotime Biotechnology, China) and subsequently transferred to a polyvinylidene difluoride membrane (Millipore, USA). The membranes were blocked with 5% bovine serum albumin (BSA) (Solarbio Science & Technology, China) for 1 h at room temperature and incubated overnight at 4°C with primary antibodies against ALP (1:1,000; DF6225, Affinity Biosciences, Cincinnati, OH, USA), osteocalcin (OCN) (1:1,000; DF12303, Affinity Biosciences, Cincinnati, OH, USA), runt-related transcription factor 2 (RUNX2) (1:2,000; AF5186, Affinity Biosciences, Cincinnati, OH, USA), VEGF (1:1,000; AF5131, Affinity Biosciences, Cincinnati, OH, USA), GAPDH (1:5,000; T0004, Affinity Biosciences, Cincinnati, OH, USA), and β-actin (1:5,000; T0022, Affinity Biosciences, Cincinnati, OH, USA). The membranes were then incubated with secondary antibody (1:5,000; S0001, Affinity Biosciences, Cincinnati, USA). Finally, the membranes were visualized with enhanced chemiluminescence reagent (Beyotime Biotechnology, China). The band intensity was quantified using Image Lab (Bio-Rad, Hercules, CA, USA).

### VEGF Analysis by ELISA

Commercial ELISA kit for VEGF (Cusabio, Wuhan, China) was used to determine the concentrations of VEGF in the supernatant liquid from different groups following the manufacturer’s protocols.

### Immunofluorescence

BMSCs were fixed with 4% PFA and permeabilized with 0.1% Triton X-100 in phosphate-buffered saline (PBS) containing 5% BSA. After blocking with 5% BSA for 1 h, the cells were stained overnight at 4°C with primary antibodies. Subsequently, the cells were incubated with a fluorescein isothiocyanate-conjugated secondary antibody (1:1,000; ab6717, Abcam, UK) for 1 h and then stained with 4′,6-diamidino-2-phenylindole. The fluorescence signal was captured using a fluorescence microscope (DMi8, Leica, Germany).

### Angiogenesis-Related Assays *in vitro*

To further assess the angiogenic capability of melatonin, the BMSCs were treated with or without 100 nM melatonin, and the conditioned mediums were harvested after 7 days of osteogenic induction, which were used for the following assays. Subsequently, human umbilical vein endothelial cells (HUVECs) (Procell Life Science &Technology Company, Wuhan, China) were cultured and treated under different conditions (1): fresh medium (2), fresh medium with 100 nM melatonin (3), conditioned medium from BMSCs without melatonin, and (4) conditioned medium from BMSCs with 100 nM melatonin. Then, scratch wound assay, transwell migration assay, and tube formation assay were further detected as will be detailed in the following discussion.

For the scratch wound assay, HUVECs were seeded at a density of 2 × 10^5^/well in a 6-well plate. The cells were scratched after confluence under an inverted microscope (Nikon; Tokyo, Japan). Then, the cells were cultured in the aforementioned mediums. The wound images were obtained immediately and at 12 h later. The width of the wounded areas (%) was calculated as (*A*_0_ – *A*_n_)/A_0_ × 100, where *A*_0_ and *A*_n_ represent the initial wound area and the residual wound area at the metering point, respectively.

For the transwell migration assay, HUVECs were suspended and loaded into the top chamber of a transwell plate (Corning, NY, USA). The medium from the treated BMSCs was then added to the chamber. After 12 h, the unmigrated HUVECs in the upper chambers were removed by wiping the top of the membranes. The migrated cells were fixed in 4% paraformaldehyde, washed with PBS solution, and then stained with 0.5% crystal violet for 10 min. The cells were imaged and counted under the random fields of the microscope (Nikon; Tokyo, Japan).

For the tube formation assay, HUVECs were seeded into a Matrigel-coated 96-well plate at a density of 5 × 10^3^/well, Then, the cells were incubated in the aforementioned medium. After incubation for 8 h, HUVEC tube formation was observed under an inverted microscope (Nikon; Tokyo, Japan). The number of tubes was calculated by Image-Pro Plus software.

### Animal Experiments in Ovariectomized Rats

#### Ethics Statement

All experiments were approved by the Animal Care and Ethics Committee of the Southern Medical University (no. SMUL2021003), and the procedures were conducted in accordance with the policies of the Ethics Committee for Animal Research.

#### Animal Surgery and Treatment

A total of 84 female specific-pathogen-free SD rats (weight 250 ± 20 g; 12 weeks old; purchased from Zhuhai BesTest Bio-Tech Co., Ltd., Guangdong) were used in this experiment. All the rats were housed at a standard room temperature of 22 ± 2°C and humidity of 55–70% under a 12-h light/dark cycle with free access to food and water. After adaptation, 78 rats were randomly selected for bilateral ovariectomized (OVX) surgery, and 6 rats received sham surgery as previously described (29). After 3 months, 6 OVX and 6 sham surgery rats were selected for micro-computed tomography (micro-CT) and H&E staining to confirm the OVX rat model of osteoporosis. Then, 72 OVX rats were randomly selected and anesthetized for the longitudinal approach, which was performed on the medial surface of the proximal end with exposure of the proximal anteromedial metaphysis of the right tibia. Specifically, a standardized drill hole defect (3-mm diameter and 4-mm depth) was used to create a monocortical defect. After surgery, all the rats were randomly divided into three groups: low-dose melatonin treatment group (LMEL group, *n* = 24), high-dose melatonin treatment group (HMEL group, *n* = 24), and control group (CON group, *n* = 24). The LMEL and HMEL group rats were intraperitoneally injected with 10 and 50 mg/kg/day melatonin daily for 4 weeks, respectively. The CON group was injected with normal saline under the same conditions. The therapeutic dose of melatonin mentioned above was determined based on previous experiments in which melatonin showed protective effects in an OVX rat model (30, 31). The right tibiae in rats were harvested and assigned to micro-CT analysis and histological studies, which were randomly selected from each group (*n* = 6/group) at 2 weeks after tibia surgery. All the remaining rats were sacrificed, and the right tibiae were harvested at 4 weeks after treatment. Six tibia specimens were randomly selected from each group for micro-CT analysis and histological studies. The remaining tibia specimens were randomly assigned to fluorescent labeling analysis (*n* = 6 for each) and biomechanical test (*n* = 6 for each).

#### Micro-CT

Micro-CT analysis (Model μCT80, Scanco Medical Inc., Brüttisellen, Switzerland) was first used to confirm the success of the osteoporosis model. We selected the first region of interest (ROI) in the trabecular region of the tibia (1,500 μm in length and approximately 300 μm below the proximal epiphyseal plate) and reconstructed it by a computer analysis program. The histomorphometric parameters were considered as BMD, bone volume fraction (BV/TV), and trabecular number (Tb.N).

Bone repair was monitored by micro-CT at 2 and 4 weeks after tibia surgery. The central 2.5-mm-diameter region of the 3-mm-diameter defect was defined by drawing a circular contour as the second ROI to evaluate bone regeneration within the defect, which could avoid containing the native bone margins and help obtain a consistent volume of interest. After 3D reconstruction, BMD, BV/TV, Tb.N, and trabecular separation (Tb.Sp) in the ROI region were analyzed. All digitalized data and 3D images were generated by the built-in software of the micro-CT.

#### Histology and Immunohistochemical Staining

For histological and immunohistochemical (IHC) analyses, the samples were decalcified in 10% EDTA for 4 weeks after micro-CT imaging. Then, 4-μm-thick sections were then subjected to H&E staining and Masson’s trichrome staining. For IHC staining, 6-μm-thick sections were incubated with primary antibodies against OCN (1:100; DF12303, Affinity Biosciences, Cincinnati, OH, USA), VEGF (1:200; AF5131, Affinity Biosciences, Cincinnati, OH, USA), and CD31(1:200; AF6191, Affinity Biosciences, Cincinnati, OH, USA). The immunoreactivity of the analysis was determined using horseradish peroxidase detection system.

#### Sequential Fluorescent Labeling

All of the rats were intraperitoneally injected with 10 mg/kg calcein (cat. no. C0875, Sigma) at 10 and 3 days before the end of the experiment (32). At the end of the observation time (4 weeks after the treatment), the tibia samples were obtained for hard-tissue slicing and then imaged through the laser confocal microscopy (LSM 880, Zeiss, Germany). The bone mineral apposition rate (MAR, μm/d) was measured and calculated by automatic image analysis system.

#### Biomechanical Test

A three-point bending test was performed on the tibia specimens to determine the biomechanical properties by a material testing machine (ELF-3510AT, Bose, Inc., USA) as previously described (33). The bones were positioned horizontally on two supports. As the location of the bone repair area, the center of the metaphysis was positioned downward. The load and the displacement of the loading device were collected during each experiment until fracture. Data was recorded to the material testing instrument from the load–deformation curve. The maximum force at failure values (N) were recorded from the load data, and the stiffness (N/mm) was calculated as the slope of the initial linear uploading portion of the curves.

### Statistical Analysis

SPSS software version 25.0 was used for all statistical analyses. Data were analyzed by two-tailed Student’s *t*-test or analysis of variance (ANOVA), with repeated measures where applicable. Differences were determined to be statistically significant when *P*-value <0.05, with the data reported as mean ± SEM.

## Results

### Melatonin Promoted the Osteogenesis of BMSCs *In Vitro*

**Figure 1:**
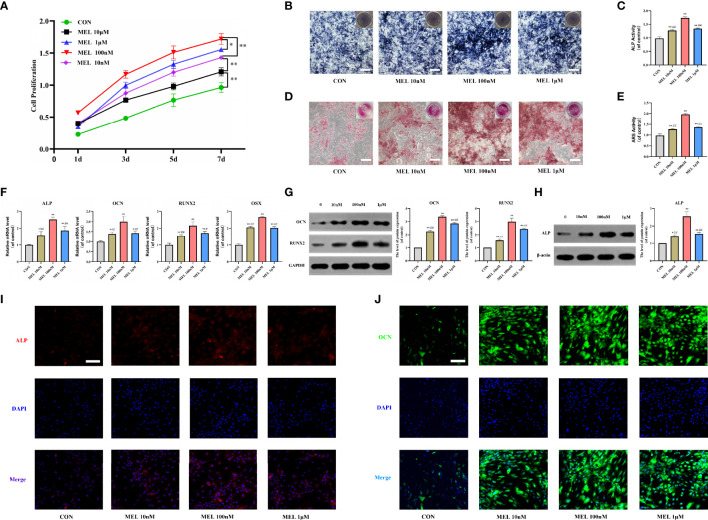
Melatonin promoted the osteogenesis of BMSCs *in vitro*. **(A)** The effect of melatonin on BMSC proliferation measured by CCK-8 assays. **(B, C)** Images and quantification of ALP activity after 7 days of osteogenic induction (scale bars, 200 μm). **(D, E)** Calcium mineralization was assessed *via* ARS staining and quantification (scale bars, 200 μm). **(F)** mRNA expression levels of osteogenesis-related markers in BMSCs following treatment with/without melatonin. **(G, H)** Protein expression levels of osteogenesis-related markers (OCN, RUNX2, and ALP). **(I, J)** Immunofluorescent images of BMSCs stained for ALP and OCN (scale bars, 100 μm). All the experiments were repeated at least 3 times independently. CCK-8, cell counting kit-8; BMSCs, bone marrow mesenchymal stem cells; ARS, alizarin red S; ALP, alkaline phosphatase; OCN, osteocalcin; RUNX2, runt-related transcription factor 2. The data are presented as means ± SEM. ^*^ *p* < 0.05, ^**^ *p* < 0.01 *vs*. control group, ^#^ *p* < 0.05, ^##^ *p* < 0.01 *vs*. 100 nM melatonin group.

First, a CCK-8 assay was conducted to observe whether melatonin can affect the proliferation of BMSCs. As shown in **Figure 1A**, melatonin promoted cell proliferation, and its effect was not in a dose-dependent manner. The most effective concentration was 100 nM, followed by 1 μM, 10 nM, and 10 μM. Then, ALP staining and ARS staining were performed to assess the pro-osteogenic effect of melatonin *in vitro*. BMSCs treated with melatonin indicated a higher level of ALP activities compared to the control group (**Figures 1B, C** **)**. The most effective concentration was also 100 nM, followed by 1 μM and 10 nM, which was consistent with the CCK-8 assay. In addition, ARS staining validated the result. After 14 days of osteogenic induction, ARS staining revealed an increase in the stained area and extracellular deposition of calcium in the melatonin treatment groups compared with the control group (**Figures 1D, E** **)**.

To further investigate how melatonin promotes the osteogenesis of BMSCs, we measured the mRNA and protein expressions of osteogenesis-related genes in cultured BMSCs at 3 and 7 days after various gradient concentrations of melatonin treatment. It showed that the mRNA expression levels of osteogenesis-related markers, including ALP, OCN, RUNX2, and osterix were all significantly increased in the melatonin treatment groups compared with the control group (**Figure 1F**). The most effective concentration is 100 nM. Consistently, the protein expression levels of ALP, OCN, and RUNX2 were all significantly increased in the melatonin treatment groups compared with the control group (**Figures 1G, H** **)**. Immunofluorescence staining also indicated higher expression levels of ALP and OCN after melatonin treatment (**Figures 1I, J** **)**. Thus, all these data indicated that melatonin promoted the osteogenesis of BMSCs *in vitro*.

### Melatonin Promoted Angiogenesis *In Vitro*

**Figure 2:**
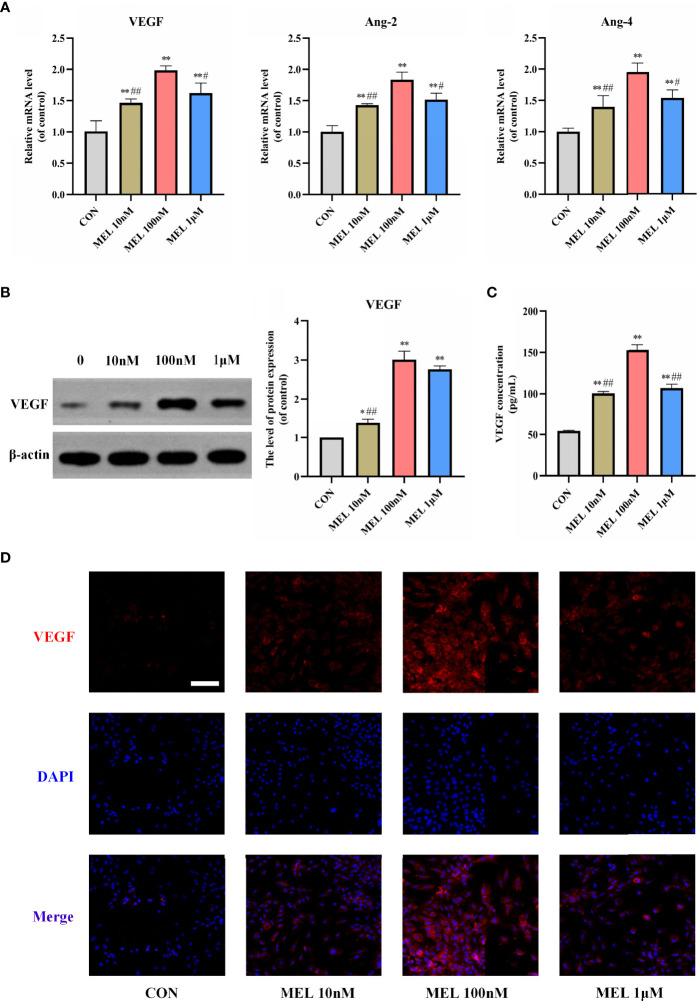
Melatonin promoted angiogenesis *in vitro*. **(A)** mRNA expression levels of osteogenesis-related markers in BMSCs following treatment with/without melatonin. **(B)** Protein expression levels of osteogenesis-related marker VEGF. **(C)** VEGF content secreted in the supernatant liquid assessed by ELISA kits. **(D)** Immunofluorescent images of BMSCs stained for VEGF (scale bars, 100 μm). All the experiments were repeated at least 3 times independently. BMSCs, bone marrow mesenchymal stem cells; VEGF, vascular endothelial growth factor. The data are presented as means ± SEM. ^*^ *p* < 0.05, ^**^ *p* < 0.01 *vs*. control group, ^#^ *p* < 0.05, ^##^ *p* < 0.01 *vs*. 100 nM melatonin group.

Then, the mRNA expressions of angiogenesis-related genes in cultured BMSCs at 3 days were detected after various concentrations of melatonin treatment. The mRNA expression levels of angiogenesis-related markers, including VEGF, angiopoietin-2, and angiopoietin-2, were all significantly upregulated compared with the control group (**Figure 2A**). After 7 days of melatonin treatment, the protein expression level of VEGF was significantly increased compared with the control group (**Figure 2B**). Consistently, ELISA and immunofluorescence staining for VEGF both showed significantly higher levels of VEGF in the melatonin-treated groups (**Figures 2C, D** **)**, Notably, the most effective concentration for all of these assays is also 100 nM. It suggested that the most effective concentration of melatonin for promoting osteogenesis and angiogenesis was consistent. These findings imply that melatonin can also promote angiogenesis *in vitro*.

### Melatonin Promoted Osteogenesis–Angiogenesis Coupling *In Vitro*

**Figure 3:**
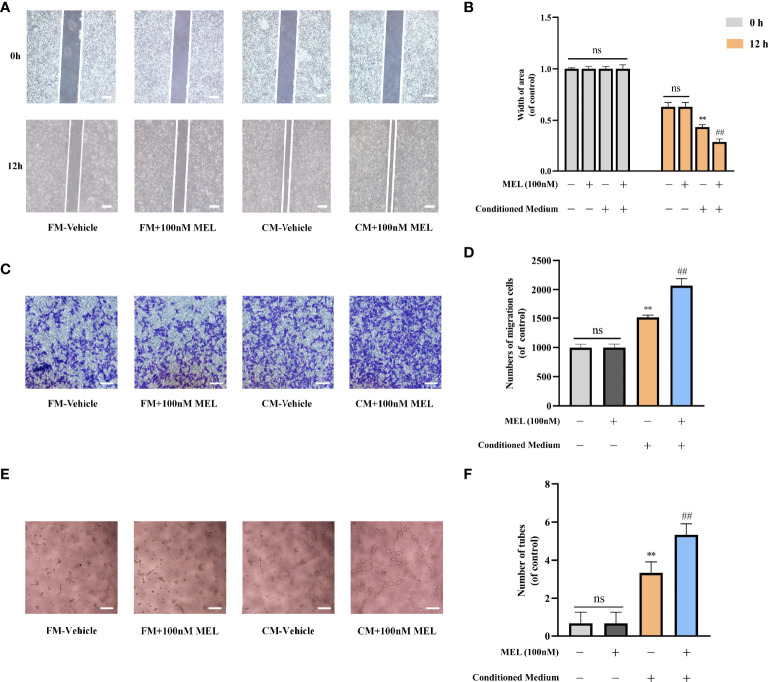
Melatonin promoted osteogenesis-angiogenesis coupling *in vitro*. **(A, B)** Scratch wound assay of HUVECs incubated with the indicated mediums (scale bars, 100 μm). **(C, D)** Transwell migration assay of HUVECs incubated with the indicated mediums (scale bars, 100 μm). **(E, F)** Tube formation assay of HUVECs incubated with the indicated mediums (scale bars, 100 μm). All the experiments were repeated at least 3 times independently. The data are presented as means ± SEM. ^**^ *p* < 0.01 *vs*. control group, ^##^ *p* < 0.01 *vs*. CM-vehicle group. HUVECs, human umbilical vein endothelial cells; FM, fresh medium; CM, conditioned medium. ns, no significance.

To further assess the angiogenic capability of melatonin, BMSCs were treated with or without 100 nM melatonin, and the conditioned mediums were harvested after 7 days of osteogenic induction. The fresh mediums and the conditioned mediums were used for the following assays, respectively. Scratch wound assay and transwell migration assay were used to explore whether melatonin could affect cell migration. We found that no significant difference in cell migration was observed between the melatonin-treated group and the control group when HUVECs were cultured in fresh medium (*P* > 0.05). However, the migration of the melatonin-treated group was incredibly increased compared to the untreated group when HUVECs were cultured in conditioned medium (**Figures 3A** **–D**). Consistently, there was no significant difference in the ability to induce capillary tube formation between the melatonin-treated group and the untreated group when HUVECs were cultured in fresh medium (*P* > 0.05). The ability of the melatonin-treated group to induce capillary tube formation was significantly enhanced compared to the untreated group when HUVECs were cultured in conditioned medium (**Figures 3E, F** **)**. We also found that the most effective concentration for the above-mentioned assays is 100 nM, which is consistent with our previous results. The results indicated that melatonin treatment was not able to facilitate HUVEC angiogenesis. However, it implied that melatonin possessed the ability to promote BMSC-mediated angiogenesis. This illustrated that the role of melatonin in promoting angiogenesis is coupled with that in promoting osteogenesis. Collectively, it demonstrated that melatonin promoted osteogenesis–angiogenesis coupling *in vitro*.

### Confirmation of Osteoporosis Model in OVX Rats

**Figure 4:**
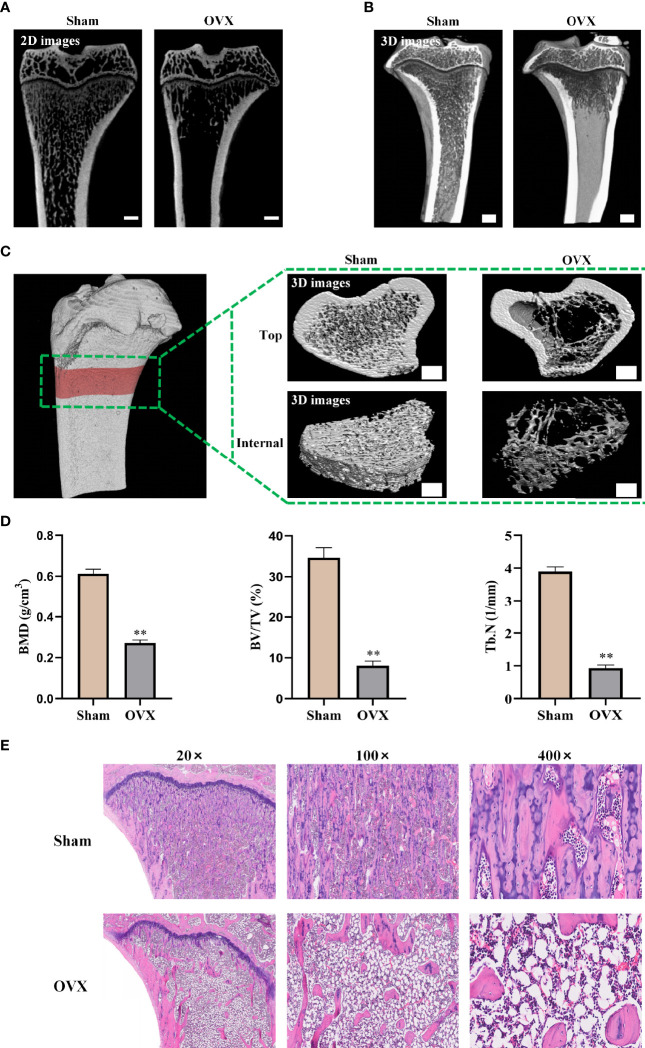
Confirmation of osteoporosis model in ovariectomized rats. **(A)** 2D images of the tibia bone in two groups (scale bars, 1 mm). **(B)** Tibia bone visualized and vertical-sectioned images (scale bars, 1 mm). **(C)** 3D constructed images of the tibia proximal metaphysis (top, trabecular bone with cortical bone; internal, trabecular portion) (scale bars, 1 mm). **(D)** Quantitative presentation of microarchitectural parameters including BMD, BV/TV, and Tb.N. **(E)** H&E staining images of tibia bone. BMD, bone mineral density; BV/TV, trabecular bone volume; Tb.N, trabecular number. The data are presented as means ± SEM. ^**^ *p* < 0.01 *vs*. Sham group.

After 3 months of OVX surgery, the efficacy of OVX was confirmed by micro-CT and H&E staining of tibia bones. 2D images and 3D vertically sectioned images of the tibia bone were performed in the Sham group and the OVX group (**Figures 4A, B** **)**. Both 2D and 3D images showed a significant trabecular bone volume, thickness, and density decrease, with a striking trabecular separation increase, compared to the Sham group. To observe this change more clearly, the 2D scanned images were constructed into 3D microstructures for analyses (**Figure 4C**). It showed significantly decreased BMD, BV/TV, and Tb.N in the OVX group compared with the Sham group (**Figure 4D**). Furthermore, H&E staining exhibited that the tibia bone in the OVX group was severely damaged, with increased bone trabecular spacing and broken tibia trabeculae, compared with the Sham group (**Figure 4E**). All of the above-mentioned data demonstrated that the OVX rat model of osteoporosis was successfully established.

### Melatonin Enhanced Bone Repairing Ability by Promoting the Osteogenesis of Tibia Defect in OVX Rats

**Figure 5:**
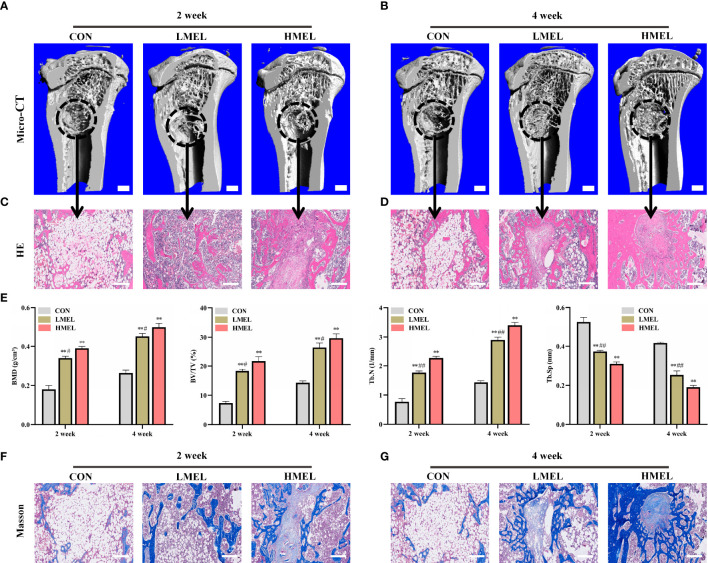
Melatonin enhanced the bone repairing ability by promoting osteogenesis of tibia defect in ovariectomized rats. **(A, B)** 3D images of mineralized bone formation in tibia defect (scale bars, 1 mm). **(C, D)** Histological assessment of the defect area by H&E staining (scale bars, 200 μm). **(E)** Quantitative presentation of microarchitectural parameters, including BMD, BV/TV, Tb.N, and Tb.Sp. **(F, G)** Histological assessment of the defect area by Masson’s trichrome staining (scale bars, 200 μm). BMD, bone mineral density, BV/TV, trabecular bone volume, Tb.N, trabecular number; Tb.Sp, trabecular separation; HMEL, high-dose melatonin treatment group. The data are presented as means ± SEM. ^**^ *p* < 0.01 *vs*. control group, ^#^ *p* < 0.05, ^##^ *p* < 0.01 *vs*. HMEL group.

After confirmation of osteoporosis, a tibia defect model was established in rats and then treated with melatonin for 4 weeks. At week 2, the CON group remained primarily empty in the 3D reconstruction images. However, a small amount of mineralized tissue was predominantly located at the defect periphery in the LMEL and HMEL groups (**Figure 5A**). At week 4, increased bone volume can be observed in the LMEL and HMEL groups compared with the CON group (**Figure 5B**). Histological representation of bone formation further confirmed the results of micro-CT (**Figures 5C, D** **)**. In addition, BMD, BV/TV, Tb.N, and Tb.Sp in the ROI were further analyzed. The CON group showed the lowest values in BMD, BV/TV, and Tb.N, with the highest value in Tb.Sp among all groups at both time points (**Figure 5E**). It indicated that melatonin treatment promoted the osteogenesis of tibia defect in OVX rats. Meanwhile, Masson’s trichrome staining showed that melatonin treatment increased the bone mineralization and formation around the tibia defect compared with the CON group (**Figures 5F, G** **)**. Remarkably, the HMEL group showed a more striking effect than the LMEL group at both time points. This suggested that melatonin therapy may show a dose-dependent manner *in vivo*. In general, these results revealed that melatonin enhanced the bone repairing ability by promoting the osteogenesis of tibia defect in OVX rats.

### Melatonin Accelerated Bone Repair by Promoting the Osteogenesis and Angiogenesis of Tibia Defect in OVX Rats

**Figure 6:**
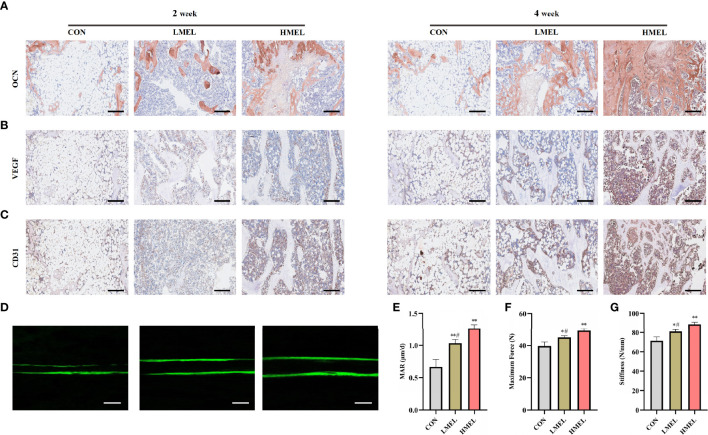
Melatonin accelerated bone repair by promoting the osteogenesis and angiogenesis of tibia defect in ovariectomized rats. **(A)** Images of immunohistochemical staining of osteocalcin in the tibia defect (scale bars, 200 μm). **(B)** Images of immunohistochemical staining of vascular endothelial growth factor in the tibia defect (scale bars, 200 μm). **(C)** Images of immunohistochemical staining of CD31 in the tibia defect (scale bars, 200 μm). **(D)** New bone formation was detected by sequential fluorescent labeling of calcein (scale bars, 10 μm). **(E)** Quantitative analysis of mineral apposition rate. **(F)** Maximum force determined experimentally by three-point bending test. **(G)** Stiffness determined experimentally by three-point bending test. The data are presented as means ± SEM. ^*^ *p* < 0.05, ^**^ *p* < 0.01 *vs*. control group, ^#^ *p* < 0.05 *vs*. high-dose melatonin treatment group.

Immunohistochemical staining of osteogenesis-related marker (OCN) and angiogenesis-related markers (VEGF and CD31) was further performed. Compared with the CON group, OCN immunostaining was denser and more widely distributed in sections in the melatonin-treated groups (**Figure 6A**), which was consistent with the results of VEGF and CD31 (**Figures 6B, C** **)**. The HMEL group showed a more striking effect than the LMEL group at both time points. Sequential fluorescent labeling showed that the distance strip in the melatonin-treated groups was wider than that in the CON group (**Figure 6D**). The bone mineral deposition rate was analyzed to investigate the bone formation activity. It showed that MAR was significantly improved in the melatonin-treated groups, and this effect was more significant in the HMEL group than that in the LMEL group (**Figure 6E**). The three-point bending test revealed that the melatonin treatment increased the ultimate load and stiffness compared with the CON group (**Figures 6F, G** **)**. All these data implied that melatonin could accelerate bone repair and increase bone strength by promoting the osteogenesis and angiogenesis of tibia defect in OVX rats.

## Discussion

The repair of bone defects requires recapitulation of complex signaling cascades, including a series of spatiotemporal angiogenesis and osteogenesis (34, 35). However, for osteoporosis, the bone resorption rate is greater than that of new bone formation, with a decreased ability for new bone formation. The clinical therapy of osteoporotic bone defect is more difficult than that of normal bone defect (36). However, conventional treatment option has limited efficacy and is not satisfactory. Therefore, novel therapeutic drug strategies to tackle osteoporosis and its related complications are warranted, which should be effective, safe, and available. To the best of our knowledge, our study is the first to demonstrate that melatonin could promote osteogenesis–angiogenesis coupling *in vitro*. Meanwhile, we further confirmed that melatonin treatment could accelerate bone repair and increase bone strength by promoting the osteogenesis and angiogenesis of tibia defect in OVX rats.

Bone regeneration is inseparable from the supply of nutrients, and angiogenesis in bone is crucial for bone defect repair (37). Vascularization is the premise of bone defect repair (38), which is a key link in the process of fracture healing and bone defect repair (39). The conventional view is that the relationship between osteogenesis and angiogenesis is one way, mainly manifested as angiogenesis providing essential nutrients for bone regeneration and repair and eliminating metabolic wastes (40). However, recent studies have shown that bone regeneration also plays a vital role in the regeneration of blood vessels within the bone (41, 42). Thus, it is critical to focus on the regeneration of blood vessels while investigating bone regeneration.

Increasing studies suggested that melatonin plays beneficial roles in bone metabolism, including bone anabolism as well as anti-bone resorption (43–45). Currently, various studies have been focused on the association between melatonin and osteogenesis. However, few studies are about the effects of melatonin on angiogenesis. In our study, we found that melatonin can promote osteogenesis and angiogenesis simultaneously. An *in vitro* study showed that melatonin promoted osteogenesis at the same optimal concentration as it promoted angiogenesis. The most effective concentration of both was 100 nM. However, it is worth noting that its effect was not in a dose-dependent manner. It indicates that it is important to find the optimal concentration, rather than simply increasing it, for melatonin to maximize its role in osteogenesis and angiogenesis. We further found that melatonin possessed the ability to promote BMSC-mediated angiogenesis and osteogenesis–angiogenesis coupling *in vitro*. When HUVECs were cultured in fresh medium, we found that no significant difference in cell migration and tube formation was observed between the melatonin-treated group and the control group. However, when HUVECs were cultured in conditioned medium, the ability of migration and induction of capillary tube formation of the melatonin-treated group was significantly enhanced compared to the untreated group. The communication between vascular endothelial cells and BMSCs was significantly critical in bone remodeling. The process can be amplified by multiple elements, which participated in the recruitment, differentiation, and proliferation of vascular endothelial cells and BMSCs (46). This association has been illustrated to be due to an osteoblastic and angiogenic factor (VEGF), which was consistent with our findings (47). Combined with the results of the VEGF-related assays in this study, it suggested that melatonin could promote BMSC-mediated angiogenesis by upregulating the VEGF levels.

Previous studies, including animal experiments and clinical findings, have shown that melatonin has a notably anti-osteoporosis effect and high safety profile (48–52). Amstrup et al. demonstrated that melatonin could improve BMD at the femoral neck in postmenopausal women with osteopenia (53). A randomized controlled trial suggested that melatonin treatment is safe in postmenopausal women with osteopenia, and small doses of melatonin can improve sleep quality (54). However, the studies on melatonin mostly focused on the effects of melatonin therapy on osteoporosis. Relative mechanisms are mainly focused on the fact that melatonin can promote osteogenesis in osteoblasts (55–57) and inhibit osteolysis in osteoclasts (58–60). There is little evidence about exploring the relationship between melatonin and angiogenesis. Ramírez-Fernández et al. showed that melatonin could promote angiogenesis in a bone defect rabbit model and may have potential beneficial effects on bone defect repair (26). Hu et al. demonstrated that melatonin could protect cortical bone-derived stem cells against γ-ray radiation and assist in the healing of postradiation bone defects (61). Yildirimturk et al. found that melatonin showed beneficial effects on the healing of bone defects in streptozotocin-induced diabetic rats (62). However, these studies lacked further clarify how melatonin affects angiogenesis and explore its effects on osteoporotic bone defect. Our study is the first to demonstrated that melatonin could promote BMSCs-mediated angiogenesis by upregulating VEGF levels and promote osteogenesis-angiogenesis coupling *in vitro*. In addition, our study is novel in showing that melatonin could accelerate bone repair by promoting osteogenesis and angiogenesis of tibia defect in OVX rats. Therefore, our study is the first to indicate that melatonin treatment was able to accelerate bone repair in rats with osteoporotic bone defect, which was potentially effective to bone regeneration. However, the limitation of our study is that the exact pathway underlying melatonin promoting osteogenesis-angiogenesis coupling has not been clarified, which is our ongoing research.

## Conclusion

In conclusion, our results demonstrated that melatonin could accelerate osteoporotic bone repair by promoting osteogenesis-angiogenesis coupling. Further investigation is undertaken about the underlying mechanism about how the osteogenesis-angiogenesis coupling process is promoted by melatonin. These findings can advance our thinking about that the application of melatonin may provide new insight and strategy for bone regeneration, and hence it could be a promising therapeutic remedy against osteoporosis and osteoporotic bone defect.

## Data Availability Statement

The original contributions presented in the study are included in the article/supplementary material. Further inquiries can be directed to the corresponding author.

## Ethics Statement

The animal study was reviewed and approved by the Animal Care and Ethics Committee of the Southern Medical University.

## Author Contributions

YL conceived and designed the experiments. SZ, CZ, and HY carried out the experiments. JL, ZF, and LL analyzed the data. All authors were involved in writing the paper and had final approval of the submitted and published versions.

## Funding

This study was sponsored by the National Natural Science Foundation of China (no. 81674095), the National Administration of Traditional Chinese Medicine TCM Inheritance and Innovation “Hundred-Thousand-Ten Thousand” Talents Project (QiHuang Scholar)–National TCM Leading Personnel Support Program (NATCM Personnel and Education Department, no. F119090038), the Innovation Team and Talents Cultivation Program of National Administration of Traditional Chinese Medicine (no. ZYYCXTD-C-202003), and the Sanming Project of Medicine in Shenzhen (no. SZZYSM202108013). The funders had no role in the study design, data collection, and analysis, or preparation of the manuscript.

## Conflict of Interest

The authors declare that the research was conducted in the absence of any commercial or financial relationships that could be construed as a potential conflict of interest.

## Publisher’s Note

All claims expressed in this article are solely those of the authors and do not necessarily represent those of their affiliated organizations, or those of the publisher, the editors and the reviewers. Any product that may be evaluated in this article, or claim that may be made by its manufacturer, is not guaranteed or endorsed by the publisher.
